# Microscopic Analysis of Degradation and Breakdown Kinetics in HfO_2_ Gate Dielectric after Ions Irradiation

**DOI:** 10.1021/acsami.5c09755

**Published:** 2025-09-04

**Authors:** Andrea Padovani, Paolo La Torraca, Fernando L. Aguirre, Alok Ranjan, Nagarajan Raghavan, Kin L. Pey, Felix Palumbo, Francesco M. Puglisi

**Affiliations:** † Department of Science and Methods for Engineering, 9306 University of Modena and Reggio Emilia, Reggio Emilia 42122, Italy; ‡ University College Cork, 261183 Tyndall National Institute, Lee Maltings, Dyke Parade, Cork T12R5CP, Ireland; § 655834 Intrinsic Semiconductor Technologies Ltd, London, U.K. EC2 V 6DN, England; ∥ Division of Nano-and-Bio Physics, Department of Physics, 11248 Chalmers University of Technology, Gothenburg 41296, Sweden; ⊥ Engineering Product Development, 233793 Singapore University of Technology and Design, Singapore 48737, Singapore; # 638301 Allegro Microsystems, Buenos, Aires 4890, Argentina; ¶ Department of Engineering “Enzo Ferrari”, University of Modena and Reggio Emilia, Via P. Vivarelli 10, Modena 41121, Italy

**Keywords:** dielectric breakdown, radiation effects, reliability, Ginestra, device simulations, high-k dielectric materials

## Abstract

We combine experiments and simulations to investigate the degradation dynamics and dielectric breakdown (BD) of SiO_*x*_/HfO_2_ gate stacks irradiated with varying doses of 40 MeV carbon ions. The analysis of postirradiation electrical characteristics (current–voltage, *I*–*V*, capacitance–voltage, *C*–*V*, and conductance-voltage, *G*–*V*) reveals that the HfO_2_ layer is the most affected by irradiation-induced damage, leading to the formation of defects consistent with oxygen vacancies. Postirradiation constant voltage stress (CVS) experiments reveal an inverse dependence of time to breakdown (*t*_BD_) and Weibull slopes (β) on the irradiation dose. These trends are accurately reproduced by statistical physics-based breakdown simulations only when accounting for partial percolation paths induced by ion strikes during irradiation, as well as a spatially correlated defect generation process during subsequent electrical stress. Our findings are crucial for designing radiation-hardened materials and improving the resilience of electronics operating in harsh environments.

## Graphical abstract

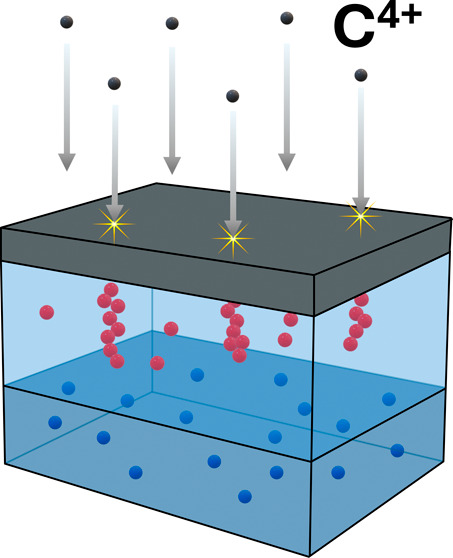

## 1 Introduction

Dielectric breakdown is a critical reliability concern in semiconductor devices and one of the most extensively studied failure mechanisms.^1^ It occurs when a dielectric—such as the gate oxide in logic or memory transistors or a low-k material in the back-end-of-line—loses its insulating properties under electrical stress, leading to the formation of a low-resistance path that compromises device and circuit functionality. Despite nearly six decades of research established a widely accepted understanding of dielectric breakdown physics, the fundamental mechanisms and their link to microscopic material properties are still only partially understood, though being key for improving device reliability.

In electronic devices operating in extreme environments—such as space technology, high energy experiments, and nuclear reactors—the scenario is further complicated by the presence of different sources of radiation.^2^ In space applications, electronic components are continuously exposed to high-energy ions from cosmic rays and solar wind. These charged particles interact with the atoms and electrons in semiconductor devices, leading to ionizing damage, atomic nuclei displacement and electron damage.^2^

Radiation-induced damage in CMOS devices has been extensively studied, primarily focusing on defect generation, increased leakage current—known as radiation-induced leakage current (RILC)^3^—and gate dielectric breakdown.^4–8^ Extensive experimental studies have shown that the substantial energy released to the lattice by impinging high-energy ions (on the order of MeV) is responsible for various phenomena, including formation of bumps in the silicon substrate,^9^ local modifications of material properties,^10,11^ creation of localized defect paths^5,6,12^ and charge accumulation^13^ in the gate dielectric layer. The extent of localized damage paths depends on the mass and energy of the incoming particles^12^ and may exhibit a discontinuous nature.^14^ While it is well established that irradiation-induced defects impact device reliability by increasing leakage current and altering time to breakdown—typically reducing it, though some studies report an increase^13^—a comprehensive understanding of the underlying physical mechanisms and their link to microscopic material properties remains elusive and has only been partially clarified with the help of simulations.^5,15–17^

In this study, we investigate the degradation dynamics and dielectric breakdown (BD) of SiO_*x*_/HfO_2_ gate stacks irradiated with varying fluences by means of experiments and simulations. Postirradiation electrical characteristics (current–voltage, I–V, capacitance–voltage, C–V, and conductance-voltage, G–V) and constant voltage stress (CVS) experiments reveal the formation of oxygen vacancy defects located mostly in the HfO_2_ layer and a reduction of both time to breakdown (*t*_BD_) and Weibull slopes (β) with increasing irradiation doses. The experimental trends are reproduced using statistical physics-based breakdown simulations while accounting for partial percolation paths induced by ion strikes during irradiation, and a spatially correlated defect generation process during subsequent electrical stress. Contrary to most works in the literature devoted to the electrical characterization of irradiated samples, that typically focus on one or two indicators (e.g., I–V curves and time-dependent dielectric breakdown, TDDB),^18^ we explore a set of four distinct electrical characteristics (I–V, C–V, G–V, and CVS). In addition, we successfully reproduce the results of all experiments in the framework of a self-consistent physics-based simulation platform that accounts for all the relevant charge transport and trapping phenomena simultaneously, including the effect of the trapped charge on the local electric field, the local thermal dissipation due to inelastic trapping at defects, and the spatially correlated nature of stress-induced defect generation in HfO_2_ (a crucial feature for the correct understanding of TDDB in this material which is seldom included in simulations). To our knowledge, this is the first time that a single, self-consistent simulation framework is used to reproduce successfully the results of multiple electrical characterization experiments on both pristine and irradiated samples, which substantially increases the confidence in the interpretation of the results and in the underlying physical picture. Thus, we believe that our findings substantially enhance the understanding of radiation-induced damage, its correlation with material properties, and its impact on dielectric degradation and breakdown, offering critical insights for the design of radiation-hardened semiconductor devices.

## 2 Experiments Methods and Materials

### 2.1 Devices and Experiments

Metal oxide semiconductor (MOS) capacitors with an area of 60 × 60 μm^2^ (which is also defined as the irradiated region, ensuring that ion exposure is confined precisely to the active device area) are fabricated on highly doped n-type silicon substrates, featuring an epitaxially grown active layer of approximately 15 μm on a 600 μm thick silicon wafer. The dielectric is a bilayer stack consisting of an unintentional ∼5 Å-thick SiO_*x*_ interfacial layer (IL) and a 65–70 Å-thick HfO_2_ high-k (HK) deposited by atomic layer deposition using H_2_O and TDMAH as gas precursors at 400 °C. A 400–500 nm-thick Al layer is used as the top electrode (TE). Dielectrics thicknesses have been estimated by means of ellipsometry measurements and transmission electron micrography (TEM).^19^

To investigate the impact of ions irradiation on degradation and breakdown, we irradiated our samples with carbon ions (C^4+^) with an energy of 40 MeV.^19^ Irradiation experiments were performed at the heavy ion microbeam facility of the Buenos Aires TANDAR Laboratory (further details regarding the facility can be found in^19–21^ ). A 20UD tandem electrostatic accelerator from National Electrostatic Corporation (NEC), capable of delivering high-energy ion beams with excellent energy resolution and long-term stability was employed. The accelerator is fed by a SNICS (Source of Negative Ions by Cesium Sputtering), which enables the generation of a wide range of negative ion species. For beam deflection and focusing, the system includes an Oxford Microbeams Ltd. OM55 high-strength magnetic quadrupole triplet lens, capable of producing focused ion beams with spot sizes adjustable between ∼2 μm and 1 mm in diameter. The irradiation chamber was maintained at high vacuum conditions throughout the procedure to ensure clean beam propagation and prevent scattering or contamination. To irradiate each capacitor, the ion beam was first adjusted to its smallest spot size to maximize spatial resolution. The focused beam was then scanned across the surface of the MOS capacitor to ensure uniform irradiation of the entire device area, with the bottom electrode of each device grounded and the capacitors left unbiased. Ion fluence was continuously monitored using a Si PIN photodiode detector (Hamamatsu S1223–01). The selection of ions species, energy and fluence/dose is based on simulations performed with SRIM software, typically used to simulate the interactions of ions with matter.^22,23^ We selected relatively light C^4+^ Carbon ions to minimize the possibility of causing the breakdown of the insulator.^22,24^ The extent of damage introduced into the dielectric stack is controlled by adjusting the ion fluence, which we will also refer to as the irradiation dose (ID). Fluence values of 10^11^ (dose #1), 10^12^ (dose #2), and 10^13^ ion/cm^2^ (dose #3) are used to achieve an ion-induced trap density ranging up to ≈10^18^ cm^–3^, as estimated through SRIM simulations (details reported in^19^). Lower and higher dosages have been excluded because they have respectively negligible impact or lead to unacceptable lattice damage.^24,25^ Figure 1 schematically represents the irradiation experiment and its effects on the IL/HK dielectric stacks and SiO_*x*_/Si interface considered in this work. C^4+^ ions impinge perpendicularly on the MOS structure, distributing uniformly across the entire sample surface (blue upper arrows). Initially, both the IL and the HK layers are assumed to contain randomly distributed pre-existing defects (1). Each impinging ion generates a discontinuous damage track through the oxide, introducing new defects along its path (2) and at the oxide/silicon interface (3). Eventually, the ion will stop its motion few micrometers into the Si substrate, after transferring all its energy to the lattice (4).

**Figure 1:**
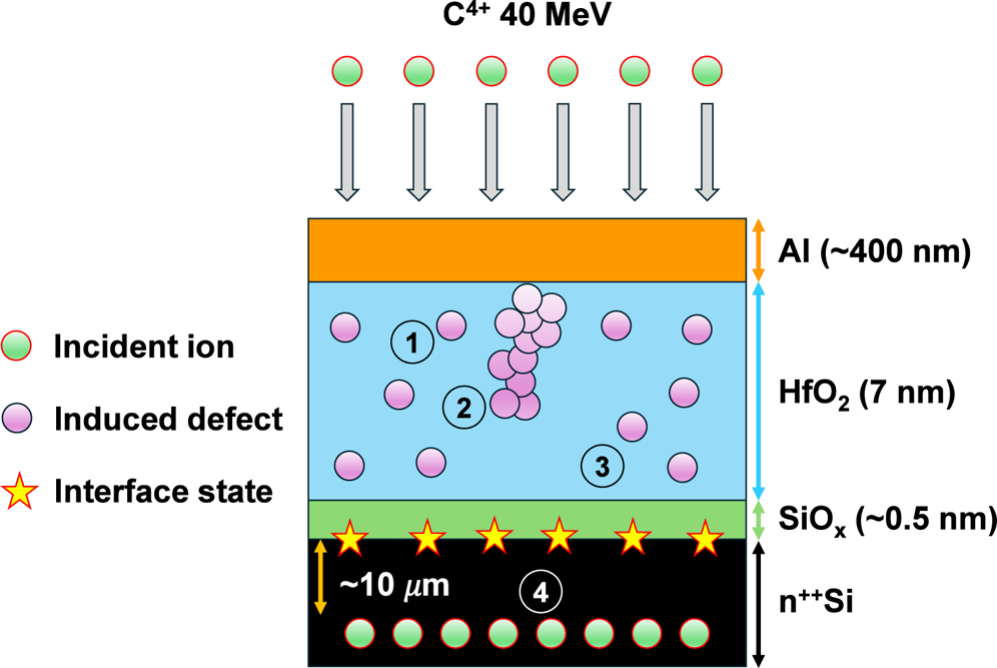
Schematic representation of an irradiation experiment and its expected effects on the dielectric stack. C^4+^ ions create new traps along their path (2) and at the Si interface (3) that sum to the pre-existing ones (1). Irradiated ions are expected to stop their motion few micrometers into the Si substrate (4).

Time-zero electrical characterization of the devices was conducted by measuring multifrequency capacitance–voltage (C–V) and conductance-voltage (G–V) characteristics, as well as leakage currents (I–V). Measurements were carried out on both fresh and irradiated devices (for all doses) to (i) monitor ion-induced degradation, and (ii) verify that they suffered no additional damage beyond the intended increase in the initial defect density. Finally, the devices were subjected to CVS at an overdrive voltage of 2.4 V with respect to flatband conditions, with a current compliance of 1 mA until hard breakdown (HBD) failure. This was done to characterize time-dependent dielectric breakdown (TDDB) distributions and analyze their dependence on irradiation. An Agilent 4285A LCR meter and a Keithley 2636B source measurement unit (SMU) were used for all the electrical tests. Electrical tests on irradiated devices were performed within a few days of exposure to ensure that the reported results accurately reflect the actual impact of the experiment.

### 2.2 Simulation Platform

Simulations are performed with Ginestra, a commercial trap-centric semiconductor device simulation platform^26^ that provides a comprehensive description of the physical mechanisms occurring in dielectrics and other materials subjected to electrical stress: carriers trapping and transport, the associated power dissipation and temperature increase, the generation of defects promoted by field, temperature and carriers injection, and the diffusion of atomic species.^27–35^

The transport of carriers through the material stack is described considering direct/Fowler-Nordheim tunneling, thermionic emission, drift-diffusion in conduction/valence and defect bands, and defect-assisted mechanisms. The latter are implemented using the theory of multiphonon trap-assisted tunneling (TAT),^27,33–35^ identified as the dominant electrical conduction mechanism in a large number of materials.^27,36–39^ Traps are treated as discrete entities characterized by two key parameters linked to their atomistic structures, the thermal ionization (*E*_T_) and relaxation (*E*_REL_) energies. In highly degraded oxides such as those at the onset of breakdown, carriers conduction occurs by means of drift through the defect bands formed by the significant amount of stress-generated traps (∼10^21^ cm^–3^).^28^

Carriers transport equations are solved in a self-consistent way together with the Poisson’s equation, the Fourier’s equations and the equations describing atomic-level material modifications induced by electrical/thermal stresses.^28,31,32^ Poisson’s equation is used to compute the potential profile within the simulated device while accounting for the applied bias, defect charge state and occupation (carriers trapping/emission processes are also described using the multiphonon theory^27^), and the metallic-like nature of any breakdown spot that may be present. The Fourier’s heat flow equation is used to calculate the profile of the temperature within the device, resulting from the external temperature and from the power dissipated during the charge transport at defect sites, defect bands and electrodes.^28^

Stress-induced material degradation is modeled accounting for multiple atomistic processes determining the creation of new defects (e.g., oxygen vacancies).^28,32,40,41^ They are implemented through an effective energy formalism^42^ computing the rate of bond-breaking (i.e., trap generation, *R*_G_) as a function of the local temperature (*T*) and of the local electric field (*F*)^28,40,41^

1 $$R_{G}=R\exp\left(-\frac{E_{A}-{\Delta E}_{corr}-p_{0}\frac{2+k}{3}F}{k_{B}T}\right)$$

the frequency prefactor *R*, the zero-field bond-breaking energy *E*_A_, and the effective dipole moment *p*_0_ depend on the degradation process considered. *k* is the material’s relative dielectric constant, and *k*_B_ is the Boltzmann’s constant. Here (1) is used in its simpler form, in which *E*_A_ and *p*_0_ are macroscopic quantities representing complex microscopic degradation processes such as the ones described in.^31,32^ In this framework, the term Δ*E*_corr_ accounts for the presence of a correlated defect generation.^40^ It represents a reduction in the energy required for bond breaking in the proximity (within bond distance, 3 Å) of an existing trap.^40^ Density functional theory (DFT) calculations suggest that *E*_A_ could indeed reduce, in these conditions, by as much as 0.7 eV depending on the charge state of the oxygen vacancy trap in HfO_2_.^43^ A value of Δ*E*_corr_ = 0.2 eV is used in this work. It is important to underline that such correlated trap generation processes have been demonstrated to play an important role especially in high-k materials such as HfO_2_ ^31,40,43^ and Al_2_O_3_.^44^

The intrinsic stochasticity of dielectric degradation and breakdown processes is considered using a kinetic Monte Carlo method to account for the randomness of the defect generation. Device-to-device variations are also included by randomly generating the spatial and energy position of pre-existing defects in every simulated device.

## 3 Results and Discussion

### 3.1 Characterization of the Dielectric Stack

To characterize the dielectric stack and assess the impact of irradiation on the electrical characteristics of the MOS capacitors under study, we measured leakage current and frequency-dependent capacitance and conductance.

Figure 2 presents the C–V and G–V curves measured before and after irradiation (at the highest dose of 10^13^ ion/cm^2^) within the 2 kHz–200 kHz frequency range, along with the corresponding simulation results. The capacitance characteristics exhibit slight frequency-dependent humps in the depletion-to-accumulation region, as shown in Figure 2a,c. Similarly, frequency-dependent peaks appear in the G–V characteristics within the same voltage range, as illustrated in Figure 2b,d. These features are typically attributed to traps at the semiconductor/dielectric interface.^45,46^ Simulations performed with the Ginestra platform (Section 2.2) reproduce simultaneously both capacitance and conductance characteristics in the whole voltage and frequency ranges. Table 1 summarizes the adopted IL and HK parameters. In this respect, the value of the relative dielectric constant for HfO_2_ used in simulations is within the range of values typically reported in the literature for its monoclinic phase,^47–50^ suggesting a relative prevalence of the latter with respect to higher-k phases (e.g., tetragonal, orthorhombic). Also, note that the 5 Å-thin IL is expected to be highly substoichiometric, with properties that are different from bulk SiO_2_.^51–53^ Indeed, by reproducing the C–V and G–V characteristics and their frequency dependence, the simulation results in Figure 2 indicate that traps at the Si/SiO_*x*_ IL interface (that is, the entire IL) are responsible for the observed frequency-dependent C–V humps and G–V peaks (listed as H-trap in Table 1). Moreover, additional traps at the Si/SiO_*x*_ IL interface with deeper energy levels (listed as deep H-trap in Table 1) are found to be responsible for the experimentally observed conductance values in the depletion region. For both trap species, the extracted E_T_ and E_REL_ values align closely with those reported in the literature for H-bridge and Hydroxyl E′ defects, as detailed in Table 2. Postirradiation experiments show a slight increase of C–V humps and G–V peaks after irradiation, as depicted in Figure 2c,d. This increase is accurately modeled by considering a small rise (20%) in the density of interface H-traps, as shown in Table 1. It is important to note that these H-related traps do not play a role in the subsequent postirradiation degradation process, as further discussed in Section 3.3. The flatband voltage (*V*_FB_) remains constant at approximately −0.23 V. These findings suggest that (i) Si/IL interface and IL are minimally affected by the irradiation and (ii) the generated traps are neutral or contribute negligible charge, as demonstrated by the C–V and G–V simulations in Figure 2 that self-consistently account for defects charge state and trapped charge. The capacitance and conductance behavior vs the applied voltage and their dispersion vs frequency in accumulation are both properly reproduced by considering the presence of two defect species in the HfO_2_ layer (labeled as VO-1 and VO-2 traps, respectively, in Table 1, where VO stands for oxygen vacancy), whose E_T_ and E_REL_ values are consistent with those estimated for oxygen vacancies.^54^ From this perspective, we speculate that the polycrystalline nature of the deposited HfO_2_ film leads to significantly broader energy level distributions for VO traps compared to density functional theory (DFT) predictions. This aligns with the relatively wide energy range used in previous studies to simulate leakage currents in HfO_2_/SiO_2_ stacks via oxygen vacancy defects.^27^ In the present study, accurately reproducing the accumulation behavior of both C–V and G–V curves was achieved by modeling this effect through two distinct trap distributions (VO-1 and VO-2) that are adjacent in energy and have different density values.

**Figure 2:**
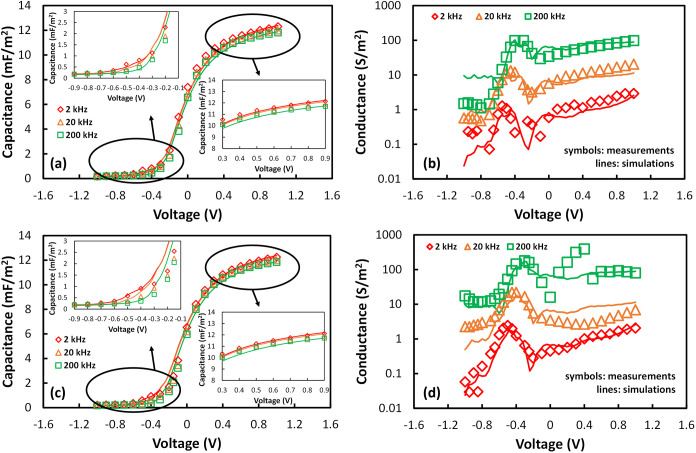
Capacitance and conductance (symbols) measured and (lines) simulated on n-Si/SiO_*x*_(5 Å)/HfO_2_(65 Å)/Al MOS capacitors (a,b) before and (c,d) after irradiation (highest dose). The insets in (a,c) show the comparison between (symbols) measured and (lines) simulated depletion and accumulation regions of the C–V characteristics.

**Table 1:** Main Parameters Considered in Ginestra Simulations, as Determined by Reproducing the Capacitance, Conductance and Leakage Current Data Shown in Figures 2 and 4 ^a^

quantity	SiO_*x*_ IL	HfO_2_	electrical data
relative dielectric constant (κ)	7.5	15	C–V
electron affinity (χ_e_)	2.05 eV	1.9 eV	I–V
band gap (*E*_G_)	6.3 eV	6 eV	I–V
electrons tunneling effective mass (*m*_te_)	0.5 m_0_	0.25 m_0_	I–V
holes tunneling effective mass (*m*_th_)	0.41 m_0_	0.25 m_0_	I–V
VO-1 ionization energy (*E*_T_)^b^		2.1 ± 0.2 eV	C–V, G–V
VO-1 relaxation energy (*E*_REL_)		1 eV	C–V, G–V
VO-1 density (N_T_) – fresh		9 × 10^17^ cm^–3^	C–V, G–V
VO-2 trap ionization energy (*E*_T_)		1.5 ± 0.4 eV	I–V
VO-2 trap relaxation energy (*E*_REL_)		1 eV	I–V
VO-2 trap density (N_T_) – fresh		4 × 10^18^ cm^–3^	I–V
VO-2 trap density (N_T_) – after 10^13^ ion/cm^2^		6 × 10^18^ cm^–3^	I–V
H-trap ionization energy (*E*_T_)^b^ ^,^ ^c^ ^,^ ^d^	2.2 ± 0.4 eV		C–V, G–V
H-trap relaxation energy (*E*_REL_)^d^	1.4 eV		C–V, G–V
H-trap density (N_T_) – fresh^d^	5 × 10^18^ cm^–3^		C–V, G–V
H-trap density (N_T_) – after 10^13^ ion/cm^2^	6 × 10^18^ cm^–3^		C–V, G–V
deep H-trap ionization energy (*E*_T_)^b^ ^,^ ^c^	3.2 ± 1 eV		G–V
deep H-trap relaxation energy (*E*_REL_)	1 eV		G–V
deep H-trap density (N_T_) – fresh	8 × 10^17^ cm^–3^		G–V
Al work function (WF)	4.05 eV		C–V, I–V

a While the C–V, G–V, and I–V data have been reproduced simultaneously to ensure consistency of the extracted values, the specific primary electrical characteristic(s) used to determine each parameter are also indicated.

b *E*_T_ is referred to the bottom of the material’s conduction band.

c *E*_T_ is scaled according to the smaller *E*_G_ and higher χ_e_ of the thin substoichiometric IL, with respect to the values of 8.9 and 0.95 eV of bulk SiO_2_.

d See also Table 2.

**Table 2:** Comparison between the Trap Ionization, *E*_T_, and Relaxation, *E*_REL_, Energies Extracted from the Simulations of the C–V and G–V Characteristics in Figure 2 with the Ones Reported for Typical Defects at the Si Interface^a^

trap	*N*_T_ (cm^–3^)^b^	*E*_T_ (eV)^c^	*E*_REL_ (eV)
this work (H-trap)	5 × 1018	2.2 ± 0.4	1.4
this work (deep H-trap)	8 × 1017	3.2 ± 1	1
H-bridge^55–57^		2.7 – 4.1	1.5 – 2.5
hydroxyl E′ center^56^		2.1–4.3	2

a The traps characterized in the IL and at the Si/IL interface align well with H-related traps.

b Due to its very small thickness of 5 Å, the entire IL is considered an interface region with a uniform volumetric defects distribution. Values refer to fresh samples.

c E_T_ values are referred to the bottom of the IL conduction band. For a direct comparison, the values of H-bridge and Hydroxyl E′ traps from^55–57^ are scaled according to the smaller bandgap (*E*_G_) and higher electron affinity (χ_e_) considered for the thin sub stoichiometric IL (Table 1), with respect to the values of 8.9 and 0.95 eV of bulk SiO_2_.

A deeper understanding of how the HfO_2_ layer is affected by the irradiation experiments can be obtained through the analysis of the gate leakage current, which is particularly sensitive to defects in the bulk of the dielectric stack.^29^ This is evident from the gate current sensitivity map (SM) reported in Figure 3 for the n-Si/SiO_*x*_(5 Å)/HfO_2_(65 Å)/Al MOS capacitor under study. It is a theoretical construct that identifies the energy-space regions of the dielectric stack where defects contribute to 95% of the total current at a specific voltage and temperature.^29^ Figure 3 shows that within the gate voltage range of interest (0.1 to 2.5 V) the conduction through the dielectric stack is driven by traps located in the bulk of the HfO_2_ layer, with no contribution from defects located in the very thin SiO_*x*_ IL. This aspect is very important also in the interpretation of degradation and breakdown, as discussed in Section 3.3. Figure 4 shows the measured and simulated I–V characteristics before and after irradiation on the same n-Si/SiO_*x*_(5 Å)/HfO_2_(65 Å)/Al MOS capacitors as in Figure 2. As expected from the SRIM simulations, the samples suffered no radiation-induced soft breakdown.^58,59^ An evident increase in leakage current is observed at low electric fields after irradiation (inset of Figure 4), whereas the high field regime is essentially unaffected, in agreement with prior works.^4^ As expected from the SM of the I–V characteristics in Figure 3, simulations very well reproduce these irradiation-dependent features by considering a 50% increase of the defects supporting inelastic TAT in the HfO_2_ layer (VO-2 trap in Table 1). Extracted E_T_ and E_REL_ (see Table 1) match the typical values reported for oxygen vacancies.^27^

**Figure 3:**
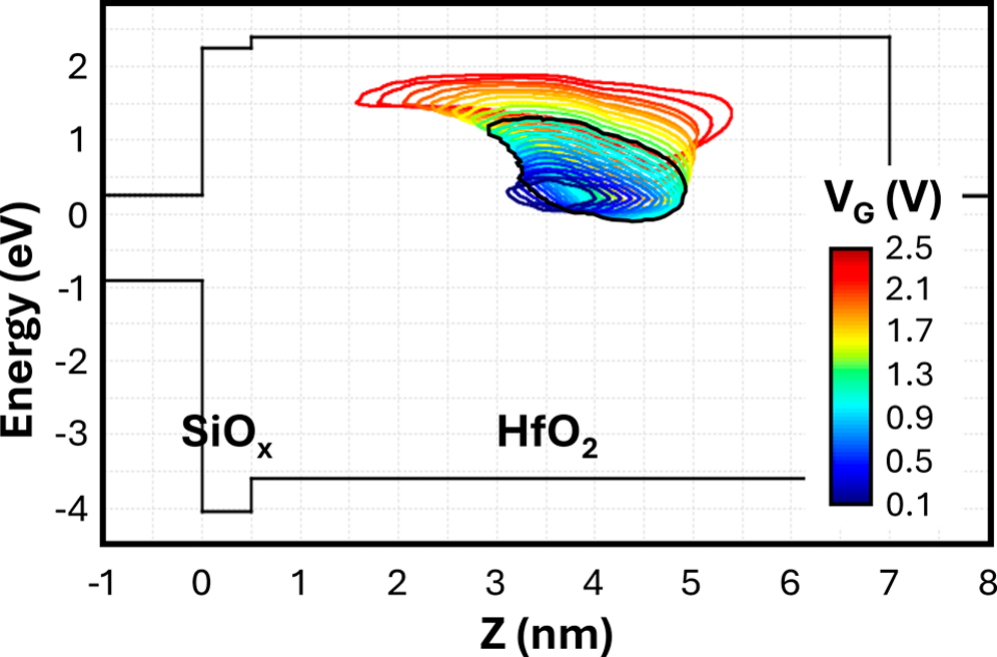
Gate current sensitivity map calculated on the n-Si/SiO_*x*_(5 Å)/HfO_2_(65 Å)/Al MOS capacitor for gate voltages from 0.1 to 2.5 V. The black contour line highlights the 0.4 to 1.2 V gate voltage (V_G_) range corresponding to the maximum postirradiation current increase as in the inset of Figure 3. For each V_G_, the sensitivity region represents the (E, z) coordinates where traps (whose properties are reported in Table 1) contribute to 95% of the total current.^29^

**Figure 4:**
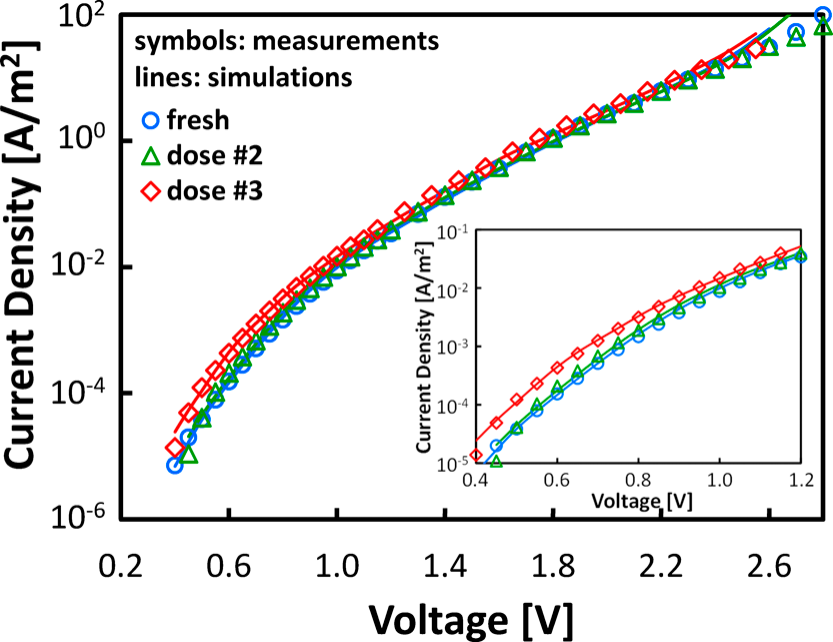
Gate leakage current densities (symbols) measured and (lines) simulated on n-Si/SiO_*x*_(5 Å)/HfO_2_(65 Å)/Al MOS capacitors before (fresh) and after different irradiation doses (dose #2:10^12^ ion/cm^2^; dose #3:10^13^ ion/cm^2^). The inset highlights the current increase in the 0.4 to 1.2 V voltage range.

### 3.2 Constant Voltage Stress and TDDB Experiments

After time-zero electrical characterization, both fresh and irradiated samples (for all doses) were subjected to TDDB testing to evaluate the impact of irradiation on degradation and breakdown. Experiments were done until hard breakdown failure by applying a CVS with an overdrive voltage of 2.4 V and a current compliance of 1 mA. Twenty-five nominally identical devices were considered in each experiment. Figure 5a shows the evolution of the gate leakage current (*I*_G_) as a function of the CVS time for nonirradiated and irradiated devices (at the higher dose of 10^13^ ion/cm^2^). I_G_ exhibits the same behavior regardless of the dose given, with a gradual initial increase (that follows a power-law trend with time typical of the so-called stress-induced leakage current phase or SILC^60^) followed by an abrupt current jump indicating the HBD event. TDDB distributions extracted from the current–time traces in Figure 5a are shown in Figure 5b on a typical Weibull plot [weibit *W* = ln­(−ln­(1 – *F*(*t*))] for all the four initial conditions considered (nonirradiated and irradiated with 10^11^, 10^12^ and 10^13^ ion/cm^2^ doses as discussed in Section 2.1).

**Figure 5:**
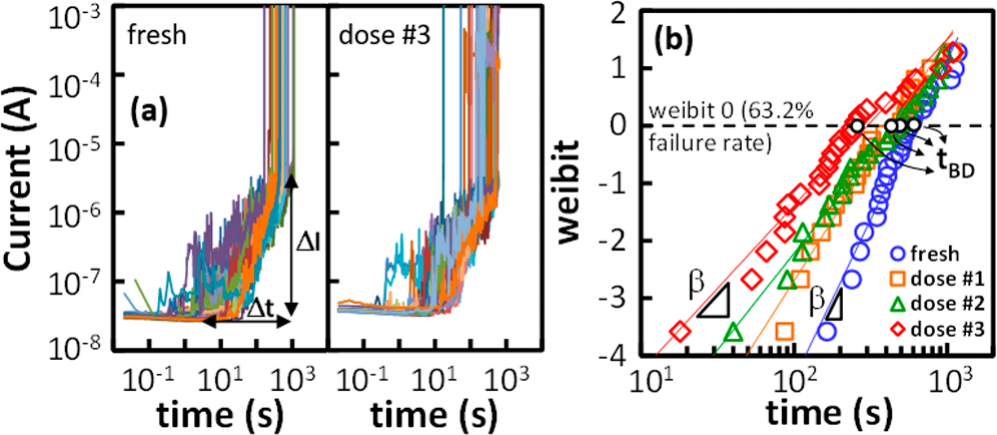
(a) Evolution of the gate leakage current measured during CVS experiments on fresh and irradiated devices (highest dose). (b) TDDB distributions (25 devices) extracted from the *I*_G_-time traces in (a) for all the irradiation doses considered (0, 10^11^, 10^12^ and 10^13^ ion/cm^2^).

To better understand and characterize how stress-induced degradation and BD are influenced by the initial irradiation conditions, we considered three figures of merit (FoMs) and monitored their dependence on the irradiation dose. The first one is the degradation slope n that is, the slope of the increase in the gate leakage current observed during the wear-out SILC phase, represented as Δ*I*/Δ*t* in Figure 5a. In this log–log current–time plot, *n* = ln­(Δ*I*)/ln­(Δ*t*) corresponds to the exponent of the power law that characterizes the SILC increase.^60^ For this reason, it is commonly referred to as the SILC exponent and serves as a clear indicator of the stress-induced degradation rate.^61,62^ The other two FoMs are the time to breakdown representing the 63.2% failure rate, t_BD_, and the Weibull slope β, both extracted from the TDDB distributions as shown in Figure 5b.

Figure 6 shows the three FoMs plotted as a function of the irradiation dose, revealing distinct signatures of the irradiation’s impact on the degradation and BD of the high-k dielectric stack under investigation. First, the degradation slope remains nearly independent of the irradiation dose, with a consistent value of approximately 0.95 across all cases [see Figure 6a]. This finding aligns with previously reported values in the literature,^63–65^ and indicates that the time dynamics of the degradation process are unaffected by irradiation, at least for the doses examined. Second, Figure 6b shows that the extracted values of *t*_BD_ and β decrease as the irradiation dose increases, dropping from 600 to 200 s and from 2.3 to 1.2, respectively. These trends can be intuitively attributed to the higher density of traps present before stress in devices subjected to greater ion fluence, as demonstrated in Section 3.1. Indeed, previous studies have shown that the Weibull slope decreases in the presence of a higher prestress defect density.^40^ This reduction occurs because fewer traps need to be generated to reach BD conditions, thereby increasing variability (reflected in a smaller β value in the Weibull plot of the TDDB data). On the other hand, while the observed decrease in *t*_BD_ with increasing irradiation dose follows a reasonable trend, its underlying mechanism is less straightforward and, to the best of the authors’ knowledge, has not been previously reported.

**Figure 6:**
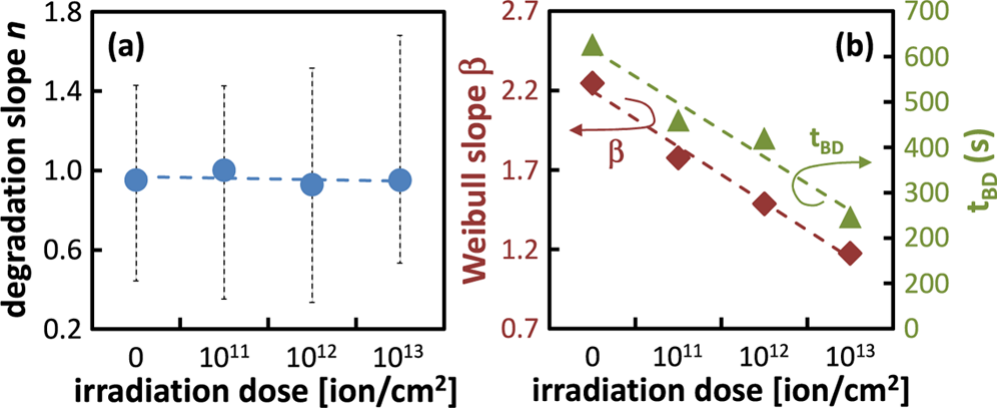
Evolution of the selected FoMs as a function of the irradiation dose, as extracted from the CVS experiments in Figure 5: (a) average degradation slope, (b) time to breakdown representing the 63.2% failure rate, and Weibull’s slope of the TDDB distribution.

In the next Section we extensively employ device simulations to investigate the physics underlying the observed dependencies of n, β and t_BD_ on the irradiation dose presented in Figure 6, providing a comprehensive and consistent explanation.

### 3.3 Simulation of Dielectric Breakdown after Irradiation

Previous implantation studies suggest that, under irradiation, traps are generated along the paths of the impinging ions as they interact with the surrounding lattice.^12,14,66,67^ The energy transfer that accompanies this interaction may be discontinuous along the incoming ion trajectory, causing intermittent damage.^14^ This is expected to result in the generation of localized damage, most probably in the shape of partial percolation paths.^10^

For what concerns the spatial–temporal dynamics of stress-induced trap generation, some important aspects must be taken into account. First, in the considered bilayer stack the SiO_*x*_ IL is highly sub stoichiometric (as discussed in Section 3.1) and is the first to break under CVS. This is confirmed also by the calculated IL/HK degradation index DI_IL/HK_ = 0.78 (according to the methodology in,^41^ with the respective IL and HK breakdown fields being 15 MV/cm and 7.25 MV/cm, and the dielectric constants reported in Table 1). This behavior arises from the voltage divider between the SiO_*x*_ IL and the HfO_2_ layer that results in the electric field in the former being much closer to its breakdown field compared to the latter. As a result, extensive bond breaking and defect generation occur early in the degradation process within the already-weak IL, while the HfO_2_ remains relatively unaffected at this stage. Once a critical density of defects forms in the SiOx, its conductivity increases substantially, leading to a voltage redistribution across the stack. This shift raises the electric field in the HfO_2_ layer, bringing it closer to its own breakdown threshold and accelerating defect generation in that region. Second, the degradation of the IL is not observable in the current–time traces measured during CVS experiments, as traps within the very thin interfacial layer do not contribute to the current, as shown in Figure 3. Consequently, the increase in *I*_G_ observed in Figure 5a is solely attributed to the generation of traps in the HfO_2_, occurring after the IL breakdown. Finally, several recent works have shown that correlated trap generation processes play an important role in high-k materials such as HfO_2_ ^31,40,43^ and Al_2_O_3_.^44^ This aspect is very important to fully understand and explain the experimental observations in Figure 6.

The different simulation scenarios that we defined to encompass all the aspects discussed above are illustrated in Figure 7. Regarding the initial irradiation, we consider either uniform damage (scenario 1) or localized damage in the form of nanoscale partial percolation paths (scenario 2) within high-k layer. The irradiation dose determines the initial number of traps present in the simulated device (before electrical stress). In Scenario 2, this leads to either a change in the number of traps within the partial percolation paths formed by ion strikes (Scenario 2′—fewer paths richer in defects) or a direct change in the number of such paths (Scenario 2″—more paths with less defects each). The initial trap density and the number of partial percolation paths considered in simulations for the different irradiation doses have been calibrated by reproducing the experimental I–V characteristics in Figure 4 while considering the different scenarios (not shown). For stress-induced degradation, we consider both a random (scenarios 1a and 2a) and a correlated (scenarios 1b and 2b) process for trap generation.

**Figure 7:**
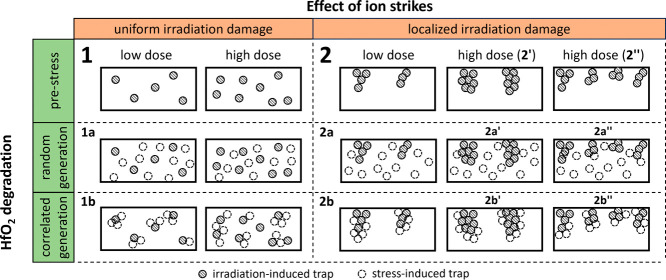
Schematic representation showing a cross-section view of HK layer for the different scenarios considered for CVS and TDDB simulations on irradiated samples: (1) uniform radiation damage with (1a) random or (1b) correlated stress-induced trap generation; (2) localized radiation damage with the formation of partial percolation paths and (2a) random or (2b) correlated stress-induced trap generation. Scenarios (2a) and (2b) are further differentiated considering that a higher irradiation dose may affect either the size of the partial percolation paths created by ion strikes [scenarios (2a′) and (2b′)], or their number [scenarios (2a″) and (2b″)].

For each case shown in Figure 7, we conducted statistical simulations (200 devices) of CVS experiments at an overdrive voltage of 2.4 V until hard breakdown using the defect-centric semiconductor device simulation platform Ginestra^26^ (see Section 2.2). It is important to note that since scenarios 2′ and 2″ in Figure 7 yield very similar results, we will henceforth refer to scenarios 2a and 2b to collectively represent 2a′/2a″ and 2b′/2b″, respectively. Results are shown in Figure 8. From the simulated current–time traces [Figure 8a–e] and TDDB distributions [Figure 8f–j], we extracted the three FoMs discussed in Section 3.2. The results are presented in Figure 9 as a function of the irradiation dose. In all simulated scenarios, the leakage current gradually increases during CVS before BD, closely matching the experimental behavior observed in Figure 5a. The extracted degradation slopes, averaged over 200 simulated devices, falls within the range of 0.95–1.2 and remain largely independent of the irradiation dose. The general trend is consistent with the experimental data presented in Figure 6a. As for β and *t*_BD_ FoMs, no changes are observed under uniform irradiation damage, regardless of whether the generation mechanism is random or correlated (scenarios 1a,b in Figure 7), which contradicts experimental findings in Figure 6b. In contrast, when partial percolation paths are considered (scenario 2 in Figure 7), both β and t_BD_ consistently decrease as the initial irradiation damage increases (higher doses).

**Figure 8:**
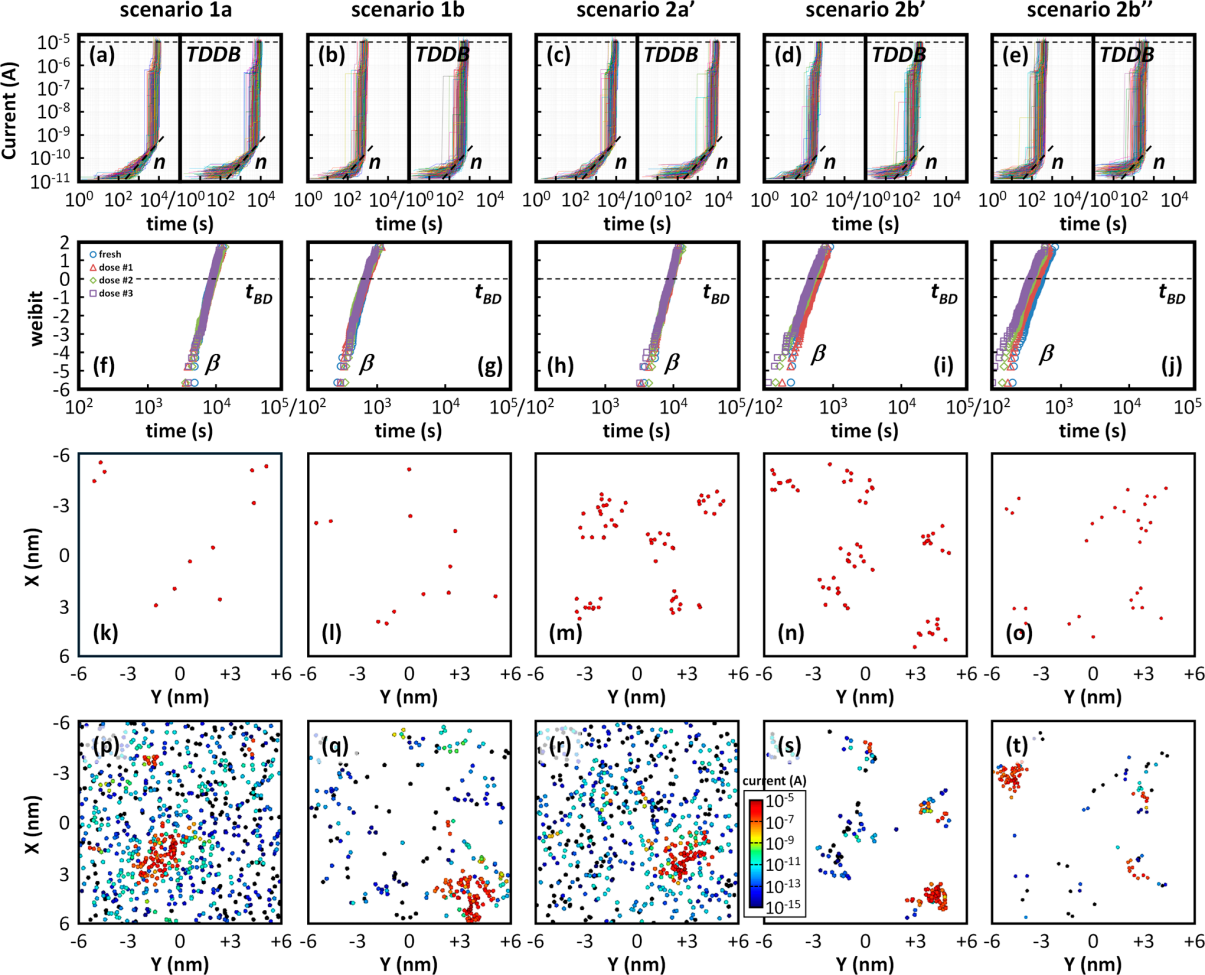
Results of statistical TDDB simulations of the different scenarios as in Figure 7. (a–e) current–time characteristics during the simulated CVS at an overdrive voltage of 2.4 V for (left) fresh and (right) irradiated devices (dose #3); (f–j) Weibull plots of the corresponding TDDB distributions; postirradiation (highest dose) 2D distributions of oxygen vacancies (spheres) in the *X*, *Y* plane (top view) considered (k–o) before stress, and (p–t) as obtained at the end of the simulated CVS experiments. In (p–t) vacancies are colored according to the current they drive: from 10^–15^ A (dark blue) to 10^–5^ A (dark red). Figures from (k–t) refer to a single representative case among the simulated statistical population of 200 devices.

**Figure 9:**
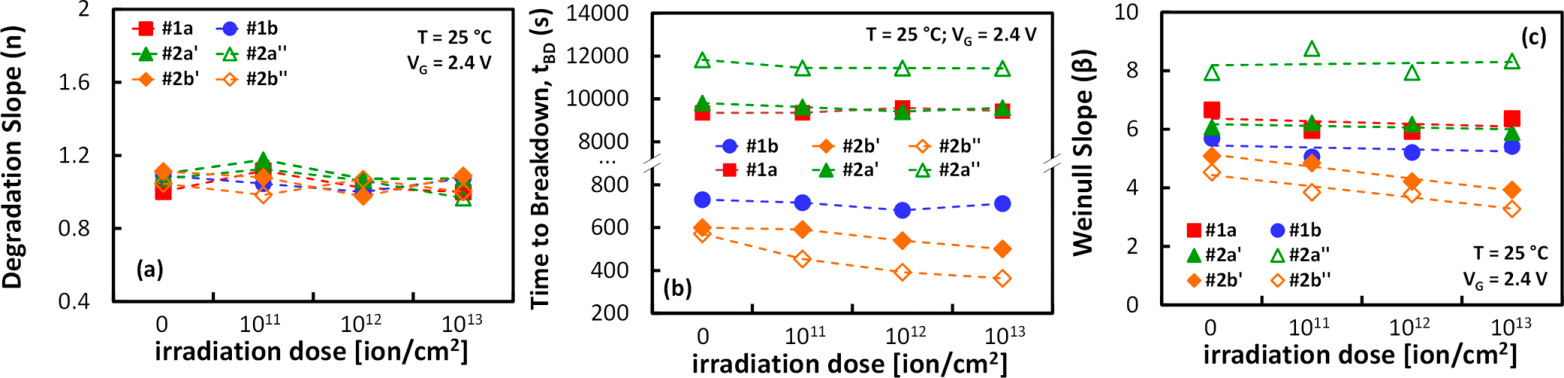
FoMs (a) *n*, (b) *t*_BD_ and (c) β as a function of the irradiation dose, as obtained from simulations of the scenarios illustrated in Figure 7. The values are extracted from simulated statistical CVS experiments at an overdrive voltage of 2.4 V (200 devices per case).

The differences in the dependence of β and *t*_BD_ on the irradiation dose under uniform or correlated stress-induced degradation can be understood by analyzing the degradation dynamics illustrated in Figure 8k–t. In discussing these results, we will introduce the term “defect cluster” to refer to a later stage of degradation occurring under electrical stress. A defect cluster represents a localized accumulation of generated traps that extends beyond any pre-existing damage and plays a critical role in triggering the positive feedback mechanism leading to dielectric breakdown.^28,68^ Although the presence of a partial percolation—formed as a result of irradiation—can accelerate the formation of a defect cluster as discussed below, the two concepts are physically distinct. The percolation path reflects a pre-existing condition, while the defect cluster develops during stress as a dynamic process leading to failure.

In the case of an uncorrelated process, defects are uniformly generated in the oxide volume. Consequently, a significant number of traps are created before a random defect cluster forms, eventually triggering the positive feedback mechanism that leads to BD.^26,68^ This holds true regardless of whether the initial conditions involve uniform irradiation damage [Figure 8k vs p] or the creation of partial percolation paths [Figure 8m vs r], though with some differences. On one hand, the described dynamics explain the overall longer breakdown times and higher Weibull slopes observed in Figure 9 for scenarios 1a and 2a. Since more defects must be generated before BD occurs, *t*_BD_ increases, while variability is reduced, leading to a higher β, consistent with.^40^ However, simulations show that in scenario 1a, the number of traps generated before BD is independent of the irradiation dose. Consequently, both β and t_BD_ are constant, Figure 9b,c. On the contrary, under scenario 2a, the number of stress-induced traps required to reach BD decreases with increasing irradiation dose. This effect, however, is relatively modest and exhibits differences between Scenarios 2a′ and 2a″. On one hand, the partial percolation paths created by irradiation enhance the likelihood of forming the defect cluster that triggers the final BD event. In fact, the BD spot typically emerges along one of these initial paths, as shown in Figure 8r [vs m]. As a result, higher irradiation doses lead to a reduction in *t*_BD_ (fewer traps need to be generated → faster BD). Nevertheless, the magnitude of this reduction is limited, reaching approximately 5% at the highest dose. On the other hand, β remains nearly unchanged across irradiation conditions, further confirming that the reduction in the trap density required to induce BD is minimal. Finally, both β and *t*_BD_ are consistently higher in Scenario 2a″ compared to Scenario 2a′. This suggests that a larger total number of traps is necessary to trigger HBD in Scenario 2a″. Although both scenarios begin with the same initial defect density, in Scenario 2a″ these defects are distributed over a greater number of partial percolation paths (see Figure 7). In the absence of a correlated generation mechanism, this broader distribution increases the total number of defects required to reach the BD condition.

Degradation and oxygen vacancy generation kinetics change significantly in the presence of a correlated process, as in scenarios 1b and 2b, where new traps are preferentially generated near existing ones [see Figure 8l vs 8q and Figure 8n vs 8s]. As a result, the total number of defects required to reach BD is substantially reduced. In scenario 2b, most of these defects effectively complete the partial percolation paths initially formed by ion strikes during irradiation, as illustrated Figure 8s,t. This mechanism explains the shorter breakdown times and lower Weibull slopes observed in Figure 9 for scenarios 1b and 2b. In scenario 2b, this effect becomes increasingly pronounced with greater initial damage, leading to a decrease of both t_BD_ and β as the irradiation dose increases, as shown in Figure 9b,c. This is because, under a correlated generation mechanism, each stress-generated defect is more likely to contribute to the formation of a viable BD path. As a consequence, the breakdown process becomes not only faster (i.e., smaller *t*_BD_) but also more variable (i.e., lower β).

The discussed simulation results provide valuable insights into the nature of damage caused by ion strikes and the underlying physics governing degradation and breakdown in irradiated devices. These findings are summarized in Table 3, which highlights the consistency of the key figures of merit (*n*, *t*_BD_ and β) across all simulated scenarios in Figure 7 with the corresponding experimental data in Figure 6. Notably, while there are differences in the magnitude of the Weibull slope—likely attributable to the additional sources of variation not included in our simulations, such as oxide thickness fluctuations and surface roughness—scenario 2b consistently explains all experimental trends. Scenario 2a also provides a close match, accurately reproducing the *n*/*t*_BD_ vs irradiation dose trends, but it does not capture the β vs ID behavior. Results in Table 3 strongly suggest that (i) radiation-induced damage leads to the formation of partial percolation paths; (ii) these paths undergo further degradation under subsequent CVS due to spatially correlated defect generation near pre-existing defects, eventually evolving into the final breakdown spot. Two additional remarks are provided here regarding the modeling approach and the robustness of the simulation results. First, the concept of partial percolation paths, as used in this work, represents an interpretive model supported by the consistency of our simulation results and established ion–material interaction physics,^5,6,10,12,14^ but it has not yet been experimentally verified. While such experimental validation would be valuable, it is beyond the scope of this study and would pose significant challenges due to the subnanometer scale and highly localized nature of the irradiation-induced damage. Second, while simulation-based results are inevitably influenced by the choice of material and trap parameters, the values used in this work are subject to stringent physical and experimental constraints. Material parameters (Table 1) fall within well-established ranges from the literature for SiO_2_ (including substoichiometric forms) and HfO_2_, ensuring physical consistency. Trap parameters are extracted through self-consistent fitting across multiple experimental observables—namely, capacitance, conductance, and leakage current curves—measured at different frequencies and irradiation levels. This comprehensive matching approach significantly restricts the viable parameter space. Furthermore, in modeling TDDB behavior, the three primary figures of merit—time-to-breakdown, Weibull slope, and degradation slope—are interdependent. Adjustments to one parameter necessarily affect the others, naturally constraining the parameter set. Although a formal sensitivity analysis is not included, the robustness of the simulation framework is supported by its ability to reproduce multiple experimental trends across varying irradiation conditions with a consistent set of parameters.

**Table 3:** Consistency of the FoMs Extracted for the Simulated CVS Experiments in Figure 8 against the Experimental Ones in Figure 6 ^a^

FoM	Scenario					
	1a	1b	2a′	2a″	2b′	2b″
degradation slope, *n*	Y	Y	Y	Y	Y	Y
time to breakdown, *t*_BD_	N	N	Y/N	Y/N	Y	Y
Weibull slope, β	N	N	N	N	Y	Y

a “Y” (“N”) indicates that the simulated FoM is (is not) consistent with the experimental one. “Y/N” is used for the *t*_BD_ of scenarios 2a′ and 2a″ because, although the simulations exhibit the expected trend, the dependence on the irradiation dose is quite small.

In future endeavors, a few aspects related to the interplay between irradiation damage and CVS conditions, such as the effect of different temperature and voltage during CVS, the effect of different irradiation conditions, and the possible different effects of irradiation on CVS (either reduction^16^ or extension^13^ of the TDDB lifetime) might be explored to extend the completeness of the understanding of the phenomena studied in this contribution. For instance, the environment temperature and the applied voltage during CVS are known to play a role in exponentially facilitating the microscopic mechanisms that are involved in TDDB, such as charge trapping and bond breaking. These effects are in fact quite well-known^1^ together with their dependence on the initial defect distribution (which may or may not result from exposure to radiation) and have been studied in detail in recent contributions. Both a higher temperature and a higher voltage during CVS result in a predictable shortening of TDDB lifetime.^69^ Nevertheless, it could be interesting to provide experimental confirmation on postirradiation samples. Furthermore, the effect of different irradiation conditions (such as ion type and energy) may be explored to provide further clarifications on the possible interplay between irradiation effects and TDDB dynamics. However, in light of the results in this study, the postirradiation degradation mechanism is not expected to fundamentally depend on the specific ion type or energy used. Still, the extent and time scale of the TDDB degradation are expected to vary depending on these parameters, since heavier ions or higher energies would result in a greater initial defect density, while lighter ions or lower energies would induce less damage. Only in extreme cases—such as very heavy, high-energy ions causing dense track damage, or very light, low-energy ions producing negligible displacement—the postirradiation TDDB behavior may differ significantly from the one reported in this study. This is also the reason according to which we selected relatively light C^4+^ (carbon) ions at 40 MeV (to intentionally minimize the risk of catastrophic damage such as insulator breakdown), relying on the guidance of SRIM simulations for the choice of ion species, energy, and dose. We therefore believe that our approach ensures that the observed behavior reflects a broadly applicable mechanism relevant to moderate ion-induced degradation, while maintaining experimental reproducibility and interpretability. Finally, conflicting results in the literature, such as reports of increased breakdown time after irradiation,^16^ definitely need a refined explanation. However, we believe that the proposed approach can be an effective starting point to possibly reconcile these alleged discrepant results. In our opinion, a primary role is played by the microscopic nature of the defects created upon irradiation and their charge state. Indeed, the effective charge of the defect perturbs the electric field in its surroundings, as recently confirmed by detailed studies on Random Telegraph Noise.^70^ Strikingly, it was recently highlighted how considering the electric field perturbations related to trapped charge in TDDB simulations can severely impact their outcomes, as local perturbations of the electric field in turn change the probability of defect generation during CVS. Finally, during CVS the charge state of a defect can change due to charge trapping phenomena. Depending on the sign and magnitude of the charge state, one may have either a local reduction or a local enhancement of the magnitude of the electric field, which may reduce or extend the TDDB lifetime.^71^ Probably, a difference in the primary defect species generated during irradiation, together with the phenomenon outlined above, might be responsible for the different behavior observed in different studies.

## 4 Conclusions

We have explored the impact of radiation dosage on the degradation and breakdown of HfO_2_-based dielectric stacks by means of comprehensive electrical characterization and multiscale defect-centric simulations. Specifically, the results of all experiments, that contrary to most works in the literature include multiple electrical tests (I–V, C–V, G–V, CVS and TDDB), are reproduced in the framework of a self-consistent physics-based simulation platform that considers all the relevant charge transport and trapping phenomena simultaneously while accounting for partial percolation paths induced by ion strikes during irradiation, and a spatially correlated defect generation process in HfO_2_ (a crucial feature seldom included in simulations).

CVS experiments evidence clear trends as a function of the irradiation dose in SILC current increase (represented by the degradation slope n) and in time to breakdown and Weibull slope extracted from the TDDB distribution. These trends are fully and consistently reproduced by breakdown simulations only when considering the creation of partial percolation paths by ion strikes during irradiation, and a spatially correlated defect generation during electrical stress. To the best of our knowledge, this is the first time that a single, self-consistent simulation framework is used to reproduce successfully the results of multiple electrical characterization experiments on both pristine and irradiated devices. Besides providing fundamental insights on physics and kinetics of degradation in irradiated gate dielectrics, our findings are extremely relevant for the improvement and design of radiation hardened semiconductor devices.

## ∇

Present affiliation: NEOM U, 4758, Al Khuraybah, PO Box 8244, Postal Code 49643 – 9136, Kingdom of Saudi Arabia
